# Main Strategies for the Identification of Neoantigens

**DOI:** 10.3390/cancers12102879

**Published:** 2020-10-07

**Authors:** Alexander V. Gopanenko, Ekaterina N. Kosobokova, Vyacheslav S. Kosorukov

**Affiliations:** N.N. Blokhin National Medical Research Center of Oncology, Ministry of Health of the Russian Federation, 115478 Moscow, Russia; alexandr.gopanenko@yandex.ru (A.V.G.); ekkos@mail.ru (E.N.K.)

**Keywords:** neoantigens, whole-exome and RNA sequencing, peptide-MHC binding predictions, pMHC-TCR binding predictions

## Abstract

**Simple Summary:**

This review provides an overview of currently available approaches applied for neoantigens discovery—tumor-specific peptides that appeared due to the mutation process and distinguish tumors from normal tissues. Focusing on genomics-based approaches and computational pipelines, we cover all steps required for selecting appropriate candidate peptides starting from NGS-derived data. Moreover, additional approaches such as mass-spectrometry-based and structure-based methods are discussed highlighting their advantages and disadvantages. This review also provides a description of available complex bioinformatics pipelines ensuring automated data processing resulting in a list of neoantigens. We propose the possible ideal pipeline that could be implemented in the neoantigens identification process. We discuss the integration of results generated by different approaches to improve the accuracy of neoantigens selection.

**Abstract:**

Genetic instability of tumors leads to the appearance of numerous tumor-specific somatic mutations that could potentially result in the production of mutated peptides that are presented on the cell surface by the MHC molecules. Peptides of this kind are commonly called neoantigens. Their presence on the cell surface specifically distinguishes tumors from healthy tissues. This feature makes neoantigens a promising target for immunotherapy. The rapid evolution of high-throughput genomics and proteomics makes it possible to implement these techniques in clinical practice. In particular, they provide useful tools for the investigation of neoantigens. The most valuable genomic approach to this problem is whole-exome sequencing coupled with RNA-seq. High-throughput mass-spectrometry is another option for direct identification of MHC-bound peptides, which is capable of revealing the entire MHC-bound peptidome. Finally, structure-based predictions could significantly improve the understanding of physicochemical and structural features that affect the immunogenicity of peptides. The development of pipelines combining such tools could improve the accuracy of the peptide selection process and decrease the required time. Here we present a review of the main existing approaches to investigating the neoantigens and suggest a possible ideal pipeline that takes into account all modern trends in the context of neoantigen discovery.

## 1. Introduction

Cancer burden significantly impacts human health and quality of life. It is one of the leading causes of death worldwide (9.6 million deaths in 2018, according to WHO) [1]. Nowadays, there are different options to treat each type of cancer. These options include surgery, radiotherapy, chemotherapy, immunotherapy [2,3,4] and are often applied in combination to improve the outcomes. Recently developed immune checkpoint inhibitors (ICIs), such as anti-CTLA-4 and anti-PD-1/PD-L1, allowed us to achieve significant progress in the treatment of several cancer types [5,6]. Now they are an established treatment for melanoma [7,8], NSCLC (non-small-cell lung carcinoma) [9,10], renal cell carcinoma [11,12], and are believed to be effective for some other cancer types [5,6,13,14,15,16]. Notably, it was observed that the efficacy of such therapy depends on the individual patient [13,17,18], which makes it necessary to investigate biomarkers of improved response. It is currently believed that a better response to ICIs is associated with PD-L1 expression, the number of tumor infiltrated lymphocytes, etc. [13,17,18,19]. It was also noticed that the efficacy of treatment with ICIs correlates with the tumor mutation burden (TMB) [20,21]. However, this trend is only observed in particular cancer types (e.g., melanoma, NSCLC, etc.) [21]. Taking into account that TMB can apparently reflect the so-called neoantigen burden [22,23,24], one could conclude that these observations highlight the importance of neoantigens as a triggering factor for tumor immune response related to T-cell activation [25,26,27]. It is not surprising that TMB is now justifiably considered to be a potential biomarker of response to immune checkpoint blockade therapy on par with other, well-established biomarkers [13,17,18,19,28].

In general, the set of somatic mutations is specific for each individual tumor specimen, with only a small number being common across samples [27,29]. Thus, it could be expected that the set of neoantigens has the same specificity. Being presented only on the surface of tumor cells by the major histocompatibility complex (MHC), which ensures their interaction with the TCR (T-cell receptor) [30], neoantigens have high immunogenic potential because they are distinguished from peptides generated by the degradation of normal proteins and thus can be recognized by the host’s immune system as non-self, which would prevent them from inducing central and peripheral immune tolerance mechanisms [31]. It is important that in contrast to TAAs (tumor-associated antigens) such as HER2, MART-1, MUC1, and CGAs (cancer germline antigens or CTAs—cancer/testis antigens) such as MAGE (melanoma-associated antigen), NY-ESO-1 (cancer/testis antigen 1) [32,33,34], which can be expressed at low levels in a variety of healthy tissues, neoantigens are specific to tumors only. Thus targeting of neoantigens (e.g., by peptide vaccination) probably leads to less side effects associated with the targeting of TAAs and CGAs, including autoimmune toxicity related to immune activation in non-target tissues [35], cytokine release syndrome [36], and others. These properties make neoantigens the perfect target for immunotherapy. It seems logical that the facilitation of neoantigen presentation to the immune system could increase neoantigen-mediated T-cell response. One possible way to achieve this aim is by utilizing the neoantigen vaccines that were already developed and investigated in a limited number of trials for several cancer types such as high-risk melanoma [37,38], glioblastoma [39], etc., yielding promising results.

Different variants of somatic mutations that lead to the production of new proteins and, thus, to mutated peptide variants were established as the primary sources of neoantigens. Neoantigens can be a result of mutations leading to amino acid changes (SNV, single nucleotide variants), frameshift mutations such as insertions or deletions, jointly known as INDELs, that modify the protein sequence downstream of their position. SNV can also break stop codons, leading to passing through translation, which yields new protein sequences corresponding to normally non-coding regions. Another source of neoantigens is the translation of non-coding regions of the genome, such as long-noncoding RNAs [40]. Nowadays, the widespread use of so-called high-throughput techniques, mainly NGS, combined with a significant reduction in their cost, helps identify somatic mutation spectra and convert the resulting data into predictions of neoantigens. The most suitable NGS-based approaches applied for these purposes are whole-genome sequencing (WGS) and whole-exome sequencing (WES) coupled with RNA-seq. In contrast with whole-genome sequencing, WES offers a balance between costs and benefits. WES targets about 2–3% of the whole genome that is related to protein-coding genes [41] and is believed to be the main source of somatic mutations leading to the appearance of mutated proteins. Simultaneous use of RNA-seq provides information on gene expression levels, allowing to select the genes that are more likely to produce real proteins and also, in some cases, could serve as a starting point for somatic mutation calling [42,43]. Moreover, RNA-seq is capable of revealing other types of neoantigen sources, such as gene fusion [44,45], alternative splicing isoforms and RNA editing events [46]. In the absence of clinical HLA (human leukocyte antigen) typing data, RNA-seq can be an additional source for in silico typing to improve the confidence of WES-based HLA determination [47]. High-confidence somatic mutation data that were identified based on WES and RNA-seq are commonly used in the isolation and ranking (or prioritization) of mutated peptide sequences aiming to detect peptides with the highest probability of being bound to the MHC and presented on the cell surface for TCR recognition [48,49]. Most algorithms typically apply HLA-peptide binding affinity as the primary ranking parameter [50]. As mentioned above, WES and RNA-seq also provide a foundation for HLA typing [48,49,50]. The individual set of HLA alleles (HLA allotype) is also required for peptide ranking because MHC-peptide affinity strongly depends on HLA variants [51]. At present, the prediction power of HLA typing tools that extract data from WES and RNA-seq is almost equal to the gold standards of HLA typing, such as PCR [52,53]. Nevertheless, if clinical data on the HLA type are available, it is more advisable to use them [50]. Perhaps the main weakness of the NGS-based approach is the absence of direct experimental evidence of the existence of predicted neoantigens. Today, the rapid progress in high-throughput mass-spectrometry approaches and the ensuing significant increase in sensitivity and accuracy [54,55,56] allows for direct identification of HLA binding epitopes. Modern LC-MS/MS makes it possible to identify thousands of MHC-bound peptides in a single experiment compared to tens in early studies [56]. Moreover, the MS data containing information on MHC-peptide interactions could serve as a reliable source for the creation of training datasets that play a critical role for machine learning-based approaches to MHC-peptide predictions [56,57]. This is especially important since prediction algorithms are mainly trained on biochemical data that could miss the entire picture of HLA-peptide binding features. Additionally, MS allows us to reveal tumor-specific post-translational modifications [58,59] and proteasome-generated spliced peptides [60] that also could contribute to the tumor-specific antigenic landscape. Finally, structure-based approaches could also improve the accuracy of the peptide selection process [61] by helping identify the effects of the structure and physicochemical properties of peptides on their immunogenic potential. A serious limitation of such approaches is due to the need for high-resolution crystallographic models and significant computing power to implement this analysis [32]. As most neoantigens have no immunogenic properties, elaborate pipelines are required to overcome all the obstacles described above to select the best candidates. The ideal algorithm for peptide selection should be able to answer the following general questions: (1) Is the gene containing a somatic mutation transcribed and, more importantly, is its mRNA translated into a protein that can be processed by the immunoproteasome into corresponding peptides? (2) Which of the peptides could result from proteasome-mediated degradation of proteins, and which of them could reach the MHC and interact with the MHC? (3) Which of these peptides have the highest affinity to the MHC and are more likely to be presented on the cell surface? (4) Can TCRs recognize the MHC-bound neoantigen complex?

It is important to note that the approaches mentioned above require downstream data analysis tools and algorithms for obtaining reliable results that could be used in clinical practice. Significant progress was made in this field during the last decade. The accuracy of neoantigen prediction is a top priority in the process of neoantigen vaccine development. Here we attempt to give a brief review of existing methods that are used to investigate neoantigens, including genomics-based in silico predictions, MS- and structure-based approaches, and describe their possible interactions and cross-validation potential. This review does not aim to give a detailed description of the available approaches and tools that are described in numerous reviews (see, e.g., [49,50,52,61,62,63]). It is meant to provide a bird’s eye view of the main trends in the context of neoantigen identification, present interactions between different approaches and propose possible improvements.

## 2. Genomics-Based Approaches and Current Bioinformatics Pipelines 

**Figure 1:**
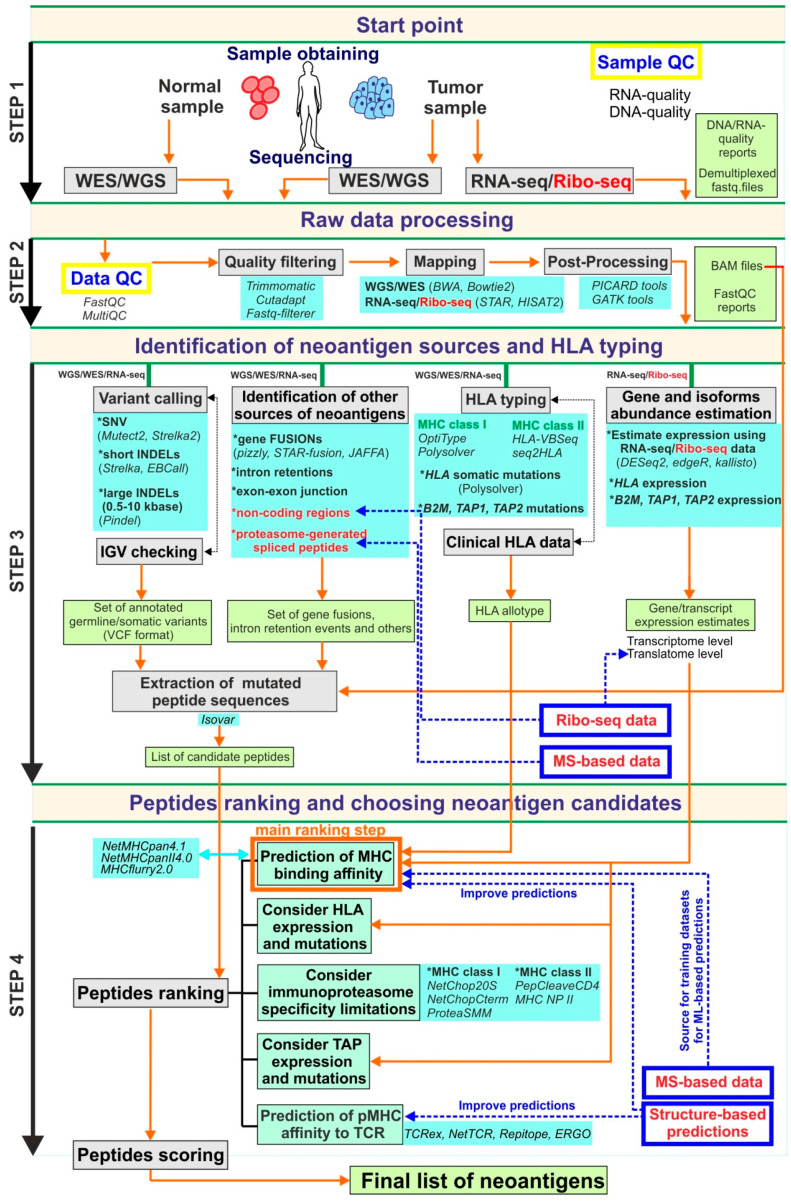
The schematic description of the possible ideal genomics-centric pipeline for neoantigen identification. In this scheme, the pipeline is formally split into four steps. Step 1 is related to sample obtaining, DNA/RNA-extraction, libraries preparation and high-throughput sequencing. Step 2 is associated with raw NGS data processing, quality-filtering and obtaining aligned to reference reads in an appropriate format suitable for downstream analysis. The aim of Step 3 is to obtain all possible information from processed NGS including all variants set, HLA allotype, expression estimations, as well as, candidate peptide sequences that further to prioritization procedures of Step 4. At the final Step 4 candidate peptide list obtaining based on identified variants used for peptide ranking using mainly MHC binding estimators. Additional options such as TCR-pMHC binding affinity scoring, TAP-transport, etc., should be considered during the prioritization step. Grey boxes are reflected main procedures that should be done in each step; light green boxes contain data that should be generated on each step; light blue boxes present possible tools that could be applied for the corresponding procedure; blue boxes with red text (as well as red text alone) are related to additional approaches that could add value to this pipeline; the orange arrows show the information flow through pipeline workflow; the blue dashed arrows highlight the steps that could be improved by additional approaches.

Currently, genomics-based strategies are some of the most promising in the field of neoantigen development. The widespread use of NGS-based techniques stimulates the development of bioinformatics tools, including those that are implemented in clinical practice. In the context of neoantigen discovery, it is important to note that the accuracy of peptide selection significantly depends on bioinformatics pipelines that are applied to the processing of the data obtained by WES and RNA-seq. A limited number of complex pipelines for these purposes were developed and described during the last several years [64,65,66,67,68]. For detailed information of selected currently available genomics-centered pipelines, see Table 1. Most of them combine principally the same set of tools (in terms of tool class) intended to carry out the main steps of analysis including raw data pre-processing to remove low-quality data, mapping to the reference genome, somatic mutation calling, mutated peptide sequences isolation and peptide ranking according to their predicted capacity to be presented on the cell surface by the MHC and recognized by the TCR. A general overview of these steps is shown in Figure 1. Current comprehensive best practices in bioinformatics in the context of neoantigen identification are presented in [50]. In this report, authors not only describe the appropriate tools for each analysis step but also provide fundamental guidelines that could serve as a basis for creating standardized consensus rules for neoantigen research.

In general, the preliminary data that can be extracted during WES/WGS and RNA-seq analysis consist of bam-files containing sequenced reads aligned to the reference genome, a set of germline/somatic mutations, the patient’s HLA allotypes, estimates of gene expression levels as well as information regarding the abundance of transcript isoforms. Somatic mutation data and RNA-seq alignments are also used to determine mutant protein sequences. 

Somatic variant identification is one of the most important and, at the same time, one of the most delicate parts of all pipelines. It is now firmly established that not only neoantigens arising from single nucleotide variants (SNV) could be candidates for vaccines. Other mutation types are also considered to be sources of neoantigens. Among them are INDELs (short insertions and deletions) [69], gene fusions [70], exon-exon junctions [71], intron retentions [72] and some other alternative splicing events [46]. RNA transcription and splicing errors [73], as well as RNA editing examples [46], could also be recognized as neoantigen sources. Non-coding genome regions such as non-coding exons, UTRs, non-coding RNAs, and others could also be neoantigen sources [74]. This list could potentially be extended by V(D)J recombination and somatic hypermutation events [75,76] that are important for blood malignancies, and sequences of viruses that are associated with some tumors [77,78]. Proteasome-generated spliced peptides, as well as peptides bearing tumor-specific post-translational modifications, could also be a source of neoantigens [60,79], but they are out of the scope of NGS-based approaches. It was reported that neoantigens resulting from non-SNV variants could make up to 15% of all neoantigens [80]. Some authors state that non-coding regions could be the main source of neoepitopes [74]. Moreover, recent proteogenomics studies of ovarian cancer revealed that the composition of tumor-specific antigens resulting from non-mutated non-exonic regions includes 29% of intronic and 22% of intergenic sequences, and most importantly, many of them are shared across tumors [81]. Thus the variety of mutation types makes it necessary to select the right tool for the identification of each type if this tool is available. Tools for the identification of some of the mutation types listed above are discussed in [50]; additionally, comprehensive comparisons can be found in [63,82,83]. Mutect2 and Strelka2 are the most reliable somatic variant callers for SNV identification [63]. It is advisable to run several somatic callers simultaneously, which could potentially improve calling accuracy [84]. It is also good practice to conduct manual verification of somatic mutation caller results by viewing them in genomic browsers and to carry out additional validation utilizing targeted sequencing approaches [50]. Identification of other neoantigen sources is also possible due to tools such as Strelka [85] and EBCall [86], which are designed for INDELs calling, and Pindel [87], which is a specialized tool for calling large INDELs. A variety of tools for gene fusion identification were also developed, such as INTEGRATE [44] (and INTEGRATE-neo pipeline [88]), STAR-fusion [45], etc. There is now a clear demand for the development of tools that can provide proper identification of all the neoantigen sources listed above.

Furthermore, after detecting all the variants of interest, one wants to know whether they could, in principle, yield a neoantigen that has a chance to bind to the MHC molecule. Firstly, it is well known that the immunoproteasome has a limited specificity, which means that not every possible mutated peptide will be produced during protein degradation [89]. Secondly, not all peptides produced by the proteasome would reach the required compartment of cells and could, in principle, interact with the MHC. It is known that before being presented by MHC class I, peptides are at first transported into the EPR (endoplasmic reticulum) by special transporters known as TAP (Transporter associated with antigen processing) and then trimmed by ER-related aminopeptidases (ERAP) [90]. There are several tools assessing TAP transport efficiency for peptides [91,92,93,94] and a number of tools that allow us to take proteasome cleavage specificity into account, such as NetChop20S, ProteaSMM [93,95] for MHC class I pathway and PepCleaveCD4, MHC II NP [96,97] for MHC class II pathway. It should also be taken into consideration that genes that code transporters of antigen-presenting machinery such as *TAP1*, *TAP2*, *B2M*, etc., can have mutations influencing their activity, and that these genes can have different expression levels in various tumor types, which has an additional impact on peptide presentation [98,99]. Thus, taking proteasome cleavage specificity and TAP transport limitations into account, the final list of peptides based on identified somatic variants should be created and subjected to subsequent prioritization procedures.

As mentioned above, currently available epitope prediction algorithms are based on the idea that the affinity of the peptide to a given MHC class molecule is the dominant contributor to neoantigen immunogenicity, and thus this parameter is considered to be the primary factor for peptide prioritization. It relies on the observation that only about 1 of 10,000 peptides resulting from protein degradation will be presented by the MHC [100]. It is also well-known that different MHC allotypes differ in specificity with respect to peptide binding. Therefore, it is crucial to know the HLA type before ranking peptides. The gold standard for HLA allotype determination is clinical HLA typing by sequence-specific PCR [101,102]. However, currently available HLA typers based on WES/RNA-seq data provide a high enough accuracy rate and can also be used for HLA allotype identification when a clinical HLA type is unavailable. Although HLA class I typing algorithms can reach an accuracy of up to 99% [103,104], HLA class II typers remain less effective and require additional development. It is no less important to estimate HLA locus gene expression as well as to determine somatic mutation patterns in this locus, as they both can be a cause of neoantigen presentation loss leading to resistance to immunotherapy [105,106,107].

Prediction of peptide-MHC binding affinity is the most critical step of the neoantigen discovery process. Many tools for such analysis exist [57,108,109,110]. These tools utilize large-scale peptide-MHC binding affinity data derived from biochemical measurements and eluted ligands data obtained by high-throughput mass-spectrometry analysis of MHC ligandome [57,111] to train machine learning-based classifiers that can identify binders and non-binders and calculate affinity scores. The machine learning approaches include linear regression (LR) and artificial neural networks (ANN). Depending on the experimental data that are used to train these algorithms, they can be classified on binding affinity (BA) trained methods, eluted ligands (EL) trained methods, and mixed trained methods utilizing both BA- and EL-datasets. Since the performance of different algorithms varies, a number of comprehensive benchmarking studies were carried out to compare the accuracy of these tools [48,49,112,113]. For instance, according to [49], where a dataset for 32 HLA class I and 24 HLA class II was used, ANN-based approaches showed better performance than LR-based, and among 19 predictors that were benchmarked, MHCflurry (AUC = 0.911 ± 0.010) and ann_align (AUC = 0.911 ± 0.004) showed the highest accuracy in terms of the AUC (Area Under ROC Curve) for MHC class I 9-mer and MHC class II 15-mer, respectively, in binding versus non-binding classification. In another benchmarking study [48], using an experimentally validated dataset with binding affinity data for 743 peptides (8- to 11-mers), derived from the HPV16 E6 and E7 proteins, none of the algorithms outperformed the others. However, different algorithms showed better performance for particular HLA types and peptide lengths [48]. In one of the most recent benchmarking studies [114], the performance of 15 algorithms was tested on a dataset described in [115], which contains 220 naturally processed vaccinia virus (VACV) peptides that were eluted from VACV-infected cells and tested for T cell immune response in infected C57Bl/6 mice. ANN-based NetMHCpan 4.0-L (AUC = 0.977), NetMHCpan 4.0-B (AUC = 0.975) and MHCflurry-L (AUC = 0.973) were reported to achieve the best performance which was in general agreement with the results previously reported in [49]. More recently, improved versions of NetMHCpan (v.4.1) and NetMHCIIpan (v.4.0) as well as MHCflurry (v.2.0) were presented [57,109]. In [57] NNAlign_MA was used to update NetMHCpan and NetMHCIIpan which outperformed the current state-of-the-art methods including NetMHCpan 4.0 and MHCflurry. O’Donnell et al. incorporated an antigen processing predictor that uses data on MHC ligands, identified by mass-spectrometry, into MHCflurry 2.0 [109], allowing it to achieve better accuracy than the currently available tools. It seems logical that the simultaneous use of several MHC-binding predictors could improve peptide prioritization. It should be noted that currently available MHC-binding predictors suffer from inadequate support for rare MHC alleles and poor performance for MHC class II molecules. Another significant inherited weakness of this approach is the failure to consider the effect of post-translational modification on binding affinity. Despite these weaknesses, this approach is the gold standard in the prediction of MHC-peptide interactions.

It is well-known that not all peptides presented by the MHC (pMHC complexes) trigger T cell activation [116,117]. For instance, in [117], the authors summarized data on candidate neoantigens predicted to be MHC-binders from 13 suitable published works, which included information about assessing the peptides’ immunogenic potential. It turned out that only 53 of 1948 neopeptide-MHC combinations elicited T cell response. In [118] it was reported that among 50 long peptides (MHC-binding prediction was performed using NetMHC 3.0) that were selected based on non-synonymous 563 somatic mutations in genes that are expressed in B16F10 murine melanoma, only one-third were immunogenic, and 60% of them elicited immune response directed against the mutated sequences. According to [119], only 25 of 66 27-mer peptides selected by predicted binding affinity to MHC I and MHC II and expression level were immunogenic according to IFNg ELISpot assay. Remarkably, in mouse models, the majority of immunogenic neoantigens (up to 90%) were associated with CD4^+^ T cell response [118,119,120]. Since the primary goal of neoantigen identification (in the context of cancer vaccines development) is to select those that would trigger or boost T-cell-mediated immune response (preferably CD8^+^ T cell response), it is essential to know which of the peptides with a high MHC binding affinity will be recognized by T-cells. This brings about the challenge of determining the specificity of MHC-epitope-TCR interactions, which could be an additional layer of the neoantigens ranking process. It is an established fact that T cells recognize pMHC complexes predominantly by the complementarity determining region 3 (CDR3) loops of the TCR [121]. Based on the fact that different individuals having different TCR repertoires can recognize the same epitopes arising from the same agents (e.g., immunodominant viral epitopes [122,123,124]), one may suggest that such epitopes have intrinsic patterns that make them more recognizable by the TCR. On the other hand, it was observed that TCR repertoires that are specific to the same epitope have similarities in their core sequences [125]. Such reasoning allows us to suggest that it is possible to perform a simulation based on sequences of peptides and TCR repertoires. Several approaches to predicting epitope-TCR binding were developed (e.g., TCRex [126], NetTCR [127], Repitope [128], ERGO [129], Deepwalk approach [130]). For instance, TCRex is based on the principle that similar TCR sequences often target the same epitope [126], Repitope is based on the idea that sequences of epitopes contain some intrinsic hidden pattern that is prone to activating T cell response [128]. Unfortunately, this class of tools is at the initial stage of development, and their prediction power suffers from insufficient training data on TCR–epitope interactions. Meanwhile, in the present time, other strategies are being successfully implemented to improve the immunogenicity of neoantigens [131,132]. Thus, in [131] the weak B16F10 neoantigens described in [118] were fused to the transmembrane domain of diphtheria toxin (DTT), significantly enhancing their ability to elicit CD8^+^ T cell response and inhibit tumor growth. A bi-adjuvant vaccine containing a neoantigen supplemented with two adjuvants such as the Toll-like receptor (TLR) 7/8 agonist R848 and the TLR9 agonist CpG, boosted the immunogenicity of the neoantigen due to efficient co-delivery and synergism of adjuvants [132].

At present, NGS techniques seem to play the primary role in the identification of therapeutic variants of neoantigens. However, the current implementation of genomics-based approaches with somatic mutations discovery as the initial step cannot answer several crucial questions. One of them is whether these mutations lead to the production of mutated proteins that can act as sources of neoantigens. It is believed that the level of mRNA does not always correlate with the protein level because not all mRNAs are translated with the same efficiency [133]. Moreover, some mRNAs are not translated at all due to being sequestered from the actively translated pool, for example, by deposition in P bodies [134]. A possible improvement that could solve this issue is based on utilizing a relatively new high-throughput technique called ribosome profiling developed in 2009 [135]. This approach involves high-throughput sequencing of ribosome-protected mRNA fragments and allows us to identify all translated mRNA, providing a snapshot of the total cellular translatome. Thus, the problem of protein production from mRNAs can be almost solved. It could also potentially reveal the translation of previously mentioned non-coding genome regions, revealing additional sources of tumor-specific neoantigens [136]. Nevertheless, genomics-based approaches are unable to solve all challenges related to the effects of post-translation modification of peptides on peptide stability and the ability to be bound by the MHC and cannot reveal proteasome-generated spliced peptide isoforms. Additionally, a lot of problems arise from highly polymorphic MHC molecules. Rare allotypes, especially MHC class II, are not supported by a sufficient volume of experimental data regarding the possibility of these types of MHC to bind peptides, making a precise ranking of neoantigens by their affinity to these MHC molecules impossible. However, integration of the above-mentioned steps into the “ideal” pipeline could significantly improve the accuracy of neoantigen prediction. Only by selecting the appropriate neoantigens can specific immune response against tumors be facilitated in clinical practice, as shown in Figure 1.

**Table 1:** The list of currently available computational pipelines for neoantigen prediction *.

Pipeline	Source, Required Input Data and Otput:	Workflow and Features:	Refs.
EpiToolkit 2015	Source: http://www.epitoolkit.de (not available) Description: Web-based pipeline focused on vaccine design. It includes simplified interfaces allowing to combine tools into a workflow. Input: Not described. Output: Interactive presentation of the results as HTML and Internal representation (List of predicted peptides with scores).	MHC genotyping (OptyType) Epitope and neoepitope prediction (SYFPEITHI, BIMAS, SVMHC, NetMHC family, UniTope, TEPTITOPEpzn) Epitope selection for vaccine design Epitope assembly	[137]
FRED2 (FRamework for Epitope Detection) 2016	Source: https://github.com/FRED-2/Fred2 Description: Computational pipeline for T-cell epitope detection and vaccine design implemented in Python. Can be extended by additional tools. Input: Sequencing reads (FASTA format). Output: Not described.	HLA typing (OptiType, Polysolver, seq2HLA, ATHLATES) T-cell epitope prediction Epitope prediction (NetMHC 3.0) TAPPrediction CleavagePrediction (NetChop) Epitope selection (OptiTope) Epitope assembly (String-of-Beads, Spacer Design)	[138]
TepiTool 2016	Source: http://tools.iedb.org/tepitool/ Description: Web-based user-friendly computational pipeline for T cell epitope prediction hosted by IEDB. It is applicable to human, chimpanzee, cow, gorilla, macaque, mouse and pig. The web-tool associated article contains a step-by-step protocol of analysis with a comprehensive description of each step, recommendations to do, and a description of anticipated results. Input: Protein sequences in single-letter amino acid code (FASTA format), the list of HLA alleles. Output: Tables with peptide sequences with predicted features.	Provide sequence data Select the host species and MHC allele class Select the alleles for binding prediction Select peptides to be included in the prediction Select preferred methods for binding prediction and peptide selection and cutoff values (for MHC class I—Consensus (IEDB recommended 2006), NetMHCpan 2.8, NetMHC 3.4, etc; for MHC class II - Consensus (IEDB recommended 2006), NetMHCIIpan 3.0, NetMHCII 2.2, etc.) Review selection, enter job details and submit data	[139]
Vaxrank 2017	Source: https://github.com/openvax/vaxrank Description: Computational framework for selecting neoantigens for vaccine peptides based on tumor mutations, tumor RNA sequencing and HLA type data. It was designed and used in the Personalized Genomic Vaccine Phase I trial (NCT02721043). Input: Tumor mutations (VCF format), tumor RNA-seq (BAM format), patient HLA alleles. Output: Set of vaccine peptides.	Determination of RNA abundance and extraction of mutated protein sequences Predicting MHC binding (MHCtools) Ranking mutant sequences Optimizing sequences for peptide synthesis	[66,67]
neoantigeneR 2017	Source: https://rdrr.io/github/tangshao2016/neoantigenR/ Description: R-based pipelines for neoantigen prediction using raw NGS data. Input: DNA-Seq, RNA-Seq, ExomeSeq (tumor and/or normal) short or long sequence reads (FASTA format), GFF annotation. Output: The list of high-affinity HLA class I binding neoantigen candidates.	Sequence alignment and isoform calling (Bowtie2, Cufflinks) Epitope prediction: extracting putative novel peptide sequences Candidate scoring by MHC binding prediction (NetMHC 3.4)	[140]
CloudNeo 2017	Source: https://github.com/TheJacksonLaboratory/CloudNeo Description: Cloud-based (implemented on CWL) workflow for neoantigen identification using NGS data. Input: VCF format (list of non-synonymous mutations), BAM format (for HLA typing). Output: HLA binding affinity predictions for all mutated peptides.	VCF processing and extraction of mutated peptide sequences (Protein_Translator) HLA typing (Polysolver, HLAminer) Peptide-MHC affinity prediction (NetMHCpan 3.0)	[141]
MuPexi (Mutant peptide extractor and informer) 2017	Source: http://www.cbs.dtu.dk/services/MuPeXI/ Description: Web-based tool for neo-epitope identification using somatic mutation calls (SNV, INDELs) and obtaining information about HLA binding affinity, expression level, similarities to self-peptides and mutant allele frequency for each mutated peptide. Supplemented by brief instructions and output format description. Input: Somatic mutation calls (VCF format), list of HLA types, gene expression profile (optional). Output: Table with all tumor-specific peptides derived from substitutions, insertions and deletions with annotation (HLA binding affinity and similarity to normal peptides).	Effect prediction (The Ensembl Variant Effect Predictor)—selecting of non-synonymous mutations Neo-peptide extraction The similarity to normal peptide estimation: removing mutated peptides similar to peptides in the human proteome from prioritization Prediction of HLA binding (NetMHCpan 3.0) Gene expression profiling Annotation Prioritization	[142]
TIminer (Tumor Immunology miner) 2017	Source: https://icbi.imed.ac.at/software/timiner/timiner.shtml (not available) Description: Computational framework that provides complex immunogenomic analysis including HLA typing, neoantigens prediction, characterization of immune infiltrates and quantification of tumor immunogenicity. Input: RNA-seq reads (FASTQ format), somatic DNA mutations (VCF format). Output: Not described.	HLA genotyping (Optitype) Prediction of tumor neoantigens (NetMHCpan 3.0) Characterization of tumor-infiltrating immune cells from bulk RNA-seq data (kallisto) Quantification of tumor immunogenicity from expression data	[143]
TSNAD (Tumor-specific neoantigen detector) 2017	Source: https://github.com/jiujiezz/tsnad Description: Pipeline with GUI allowing to identify tumor-specific mutant proteins according to GATK best practices. It provides two strategies: 1.Extraction of extracellular mutations from membrane proteins; 2. MHC affinity prediction for class I MHC. Allows us to start from raw NGS data. Input: Pair-ended sequencing data (FASTQ format) from WES. Output: List of somatic mutations with annotations, extracellular mutations of the membrane proteins and the MHC-binding information (TXT format).	Detection of cancer somatic mutations according to GATK best practices (Trimmomatic, BWA, samtools, Picard tools, GATK tools, ANNOVAR) Prediction of neoantigens (TMHMM—for extracellular mutations, NetMHCpan 2.8—for MHC-binding affinity prediction for class I MHC).	[144]
INTEGRATE-neo 2017	Source: https://github.com/ChrisMaherLab/INTEGRATE-Neo Description: The pipeline is focused on the discovery of neoantigens derived from gene fusions. Input: Reads in FASTQ format, the human reference genome in FASTA format, gene models in GenePred format, genes fusion in BEDPE format predicted by INTEGRATE. Output: BEDPE format file.	Gene fusion peptide prediction HLA allele prediction (HLAminer) Gene fusion neoantigen discovery (NetMHC 4.0)	[88]
NeoepitopePred 2017	Source: https://github.com/stjude/NeoepitopePred Description: Workflow for identification of putative neoepitopes derived from SNV and gene fusions based on WGS data. Input: FASTQ format (PE or SE) or BAM format files, Output: Not described.	HLA typing—stjude-hlatype applet (OptiType) Predict affinity of peptides to HLA—stjude-epitope applet (NetMHCcons 1.1) Identification of Fusion junctions (CICERO)	[145]
Neopepsee 2018	Source: https://sourceforge.net/projects/neopepsee/ Description: Machine learning-based neoantigen prediction tool for NGS data. Input: Raw RNA-seq data (FASTQ format) and list of somatic mutations (VCF format), clinical HLA typing (if available) Output: mutated peptide sequences and gene expression levels, determination of immunogenic neoantigens.	Transcript isoform prediction HLA type prediction (HLAminer) MHC binding affinity prediction (IEDB-Peptide binding to MHC class I molecules) Feature calculation Immunogenicity classification (IEDB-T cell class I pMHC immunogenicity predictor)	[65]
ScanNeo 2019	Source: https://github.com/ylab-hi/ScanNeo Description: Computational pipeline for the identification of short and large indels-derived neoantigens utilizing RNA-seq data. ScanNeo consists of independent modules implementing three analysis steps. Input: RNA-seq data in BAM format. Output: Ranked set of neoantigens.	Indels discovery: duplicated reads removal (Picard tools) spliced reads removal (sambamba) realignment (BWA-MEM) indels calling (transIndel) Annotation and filtering Putative PCR slippage derived indels removal Indel annotation (Variant Effect Predictor) Germline indels removal Neoantigen prediction Indel-derived peptide sequences generation (pVac-seq) High-affinity peptides prediction (NetMHC 3.0 and NetMHCpan 3.0) Prediction results merging and filtering Note: HLA typing carries out using yara aligner and OptiType tool or HLA type provides by the user.	[146]
DeepHLApan 2019	Source: http://biopharm.zju.edu.cn/deephlapan/ Description: Deep learning approach for neoantigen prediction considering both HLA-peptide binding (binding model) and immunogenicity (immunogenicity model) of peptide-HLA complex. Input: CSV format files with head of “Annotation,HLA,peptide”. Only HLA-A,B,C alleles. Output: Binding score (ranges from 0 to 1, the probability that peptide binds with HLA), Immunogenicity score (ranges from 0 to 1; 0.5 is the threshold to select the predicted immunogenic pHLA).	The binding model for predicting the probability of the peptide being presented to the tumor cell membrane by HLA Immunogenicity model for predicting the potential of pHLA eliciting T-cell activation.	[147]
pTuneous (prioritizing tumor neoantigens from next-generation sequencing data) 2019	Source: https://github.com/bm2-lab/pTuneos Description: In silico tool to predict the immunogenicity of SNV-derived neoepitopes that consider MHC presentation and T-cell recognition ability. It is based on experimentally validated neoantigens. It contains Pre&RecNeo module—learning-based framework allowing to predict and prioritize neoepitopes recognized by T cells and RefinedNeo module—neoepitope scoring schema allowing to evaluate the naturally processed and presented neoepitope immunogenicity Input: PairMatchDNA (WES) mode accept WES and RNA-seq sequencing data (FASTQ format), VCF mode accepts VCF format file with mutation set, expression profile (e.g., obtained by *kallisto*), copy number profile (e.g., obtained by *sequenza*). Output: TSV files (snv_neo_model.tsv and indel_neo_models.tsv) containing extracted mutated peptides derived from non-synonimous SNV and INDELs and corresponding immunity score measures.	WES mode: Sequencing quality control (Trimmomatic) Mutation calling (Strelka) HLA typing (Optitype) Expression profiling (kallisto) Neoantigen prediction, filtering and annotation (NetMHCpan 4.0) VCF mode: Neoantigen prediction, filtering and annotation	[148]
NeoPredPipe 2019	Source: https://github.com/MathOnco/NeoPredPipe Description: Pipeline that provides predictions on multi-region sequence data and assessing intra-tumor heterogeneity (IHC) of the antigenic landscape of tumors. Input: Multi- or single region VCF files (with a set of somatic mutations), Patient HLA Types (optional) Output: Annotated variants, predicted neoantigens, predicted recognition potential, a summary of IHC statistics	Variant annotation (ANNOVAR) Neoantigen prediction (NetMHCpan 4.0) Peptide matching Neoantigen recognition potential	[68]
pVACtools 2020	Source: https://pvactools.readthedocs.io/en/latest/ Description: Computational toolkit allowing identification of altered peptides derived from SNV, INDELs, gene fusions and providing prediction of peptide-MHC binding for MHC class I and class II. Input: VCF format files, FASTA with peptides Output: A set of files containing information about predicted epitopes before and after the filtering process supplying information about binding affinity scores and other parameters.	Prediction of neoantigens from somatic alterations (pVACseq and pVACfuse (gene fusions)) Prediction of neoantigens for peptides in a FASTA file Prioritization and selection (pVACviz with graphics-based interface) Design of DNA and synthetic long peptide-based vaccines (pVACvector)	[64,149]
ProGeo-neo 2020	Source: https://github.com/kbvstmd/ProGeo-neo Description: Neoantigen prediction workflow that integrates genomic and mass spectrometry data. It consists of three modules: construction of customized protein sequence database, HLA allele prediction, neoantigen prediction and filtration. Input: RNA-seq data (FASTQ format), Genomic variants (VCF format), LC-MS/MS data (Raw format). Output: List of candidate peptides	HLA typing (OptiType) Identification tumor-specific antigens for NGS data (WES/RNA-seq) (BWA, GATK tools) MHC binding prediction (NetMHCpan 4.0) Verifying MHC-peptides using mass spectrometry data (MaxQuant) Checking potential immunogenicity of T-cell-recognition	[150]
Neoepiscope 2020	Source: https://github.com/pdxgx/neoepiscope Description: Neoepitope identification pipeline that incorporates germline context and considers variant phasing for SNV and indels. Requires DNA-sequencing data. Input: Set of somatic and germline mutations (VCF format), BAM files. Output: TSV file with the information of mutations and neoepitopes	VCF files preprocessing (merging somatic and germline variants) Haplotype phasing (HapCUT2) Neoepitope prediction (MHCflurry, MHCnuggets, etc.)	[80]
neoANT-HILL 2020	Source: https://github.com/neoanthill/neoANT-HILL Description: User-friendly python-based toolkit that combines several pipelines that ensure fully-automated identification of potential neoantigens with a graphical interface. It allows starting from raw NGS data as well as ready-to-use variant calls. Input: Somatic variants (VCF format) and/or RNA-seq data (raw or aligned) Output: User-defined generic directory that contains variant calling data, FASTA with WT and MT sequences, predicted HLA types, gene expression estimates, tumor-infiltrating immune cells quantifications.	Expression estimation (kallisto) Variant discovery (GATK tools) HLA typing (OptiType) Tumor-infiltrating immune-cell estimation (quanTIseq) Variant annotation (snpEff) MHC binding affinity prediction (IEDB tools, MHCflurry)	[151]
INeo-Epp 2020	Source: http://www.biostatistics.online/INeo-Epp/antigen.php Description: User-friendly web-tool implementing T-cell HLA class I immunogenicity prediction method based on sequence-related amino acid features utilizing the random forest algorithm. Input: Candidate peptide sequences (8-12 aa recommended), HLA allotype Output: Table containing peptides sequences annotated with score, %rank and prediction.	Providing peptide sequences and HLA types. Annotation of peptides with score metrics. Selecting immunogenic peptides with a score > 0.5 as recommended.	[152]

* The descriptions of the pipelines presented in the table are based on information provided in associated articles and obtained from the web-based source descriptions that are available on source websites. It is limited by highlighting the main features that distinguished the pipelines from each other. The date of the pipeline appearance is based on the publishing date of the supported article if other information is not provided. The source link is cited as “not available” if the website was not available at the time of writing. The output and input descriptions are presented as described in supporting articles or web-based sources (if available). In cases where a clear description was lacking, these fields were cited as “Not described”. “Workflow and features” field contains information on the main steps that are available within the workflow. The main tools utilized as a part of the described workflows are also provided if they are described in supporting articles or in web-based sources.

## 3. Mass Spectrometry-Based Approaches

Genomics-based approaches represent the gold standard that is applied for neoantigen vaccine development, including in silico peptide prediction. Neoantigen candidate selection relies on the spectra of somatic mutations identified by WES/RNA-seq. This approach suffers from a lack of direct experimental evidence of the real presence of predicted epitopes on the cell surface as a complex with MHC molecules [153,154]. Lacking data could be obtained using high-throughput mass spectrometry techniques [153,154] that at present allow us to analyze large amounts of peptides or whole proteins simultaneously. This review does not aim to give a detailed characterization of MS-based approaches; for a comprehensive review on this topic, the reader could refer to [56,153,154].

A typical MS workflow (IP-based) starts with immunoprecipitation of MHC-peptide complexes using beads conjugated with MHC-specific antibodies or beads bound with dummy antibodies as negative controls. Subsequent washing steps ensure the removal of unbound and non-specifically bound peptides, whereupon the eluted material is subjected to MS analysis. Another strategy is mild acid elution (MAE) of MHC-bound peptides from the cell surface by treatment under mildly acidic conditions [155], followed by MS analysis. This method has a significant false-positive rate and low specificity due to contamination with a large quantity of non-specific peptides. Detailed comparison of IP- and MAE-based approaches are presented in [156]. To find information on MHC peptidome identification by MS approach, the reader could refer to Zhang et al. [154] where authors provide a summary of 40 studies that were carried out from 1990 to 2019.

Unlike the genomics-based approach, which only provides for neoantigen prediction, mass-spectrometry allows us to take a real snapshot of the total MHC-bound protein interactome. Additionally, it could reveal not only neoantigens that originate from somatic mutation variants but also those which arise due to proteasome-mediated peptide splicing [157,158]. Using mass-spectrometry, it was shown that the proportion of spliced peptides relative to peptides displayed by HLA class I varies from 2-6% reported in [159] to 30% reported in [60]. Moreover, MS allows us to identify the post-translational modifications (PTM) of peptides bound to the MHC, thus shedding light on the importance of PTM for binding affinity [59]. Mass-spectrometry derived data served for the development of the first tool allowing to predict the interaction between HLA class I molecules and phosphorylated peptides [160]. In addition, MS-based profiling of HLA peptidome could generate high-quality training data that could potentially significantly improve current prediction models [57,111,161], and could also be used for benchmarking available tools.

Nevertheless, MS also has some limitations. They include low sensitivity and reproducibility. These problems are especially acute for low-abundance peptides, including tumor-specific neoantigens. Moreover, the washing stages of MHC-peptide complexes during IP could result in a loss of bound peptides. These issues impose a limitation on the initial quantities of biological material. For typical experiments, 1 g of tumor tissue or anywhere from a hundred million to billions of cells are required [156]. It should also be noted that cancer cells and tumor tissues have different HLA molecules; thus, peptides that were identified from this type of material are relevant for different HLA molecules, adding the problem of specificity of the HLA ligandome.

In summary, by combining genomics-based predictions with high-throughput HLA-ligandome mass-spectrometry data, the performance of neoantigen discovery procedures could be significantly enhanced. For instance, the currently available ProGeo-neo pipeline [150] utilizes LC-MS/MS data to verify NGS-based derived neoantigen candidates.

## 4. Structure-Based Approaches

Structure-based predictions are another option that can improve the state of the art in the context of neoantigen discovery [61,162]. While the genomics-based approach utilizes sequence-based methods, the structure-based prediction is additionally capable of uncovering the significance of peptide structure and physicochemical properties, as well as the importance of post-translational modifications, such as phosphorylation [163], citrullination [164], and glycosylation [165], for peptide binding to the MHC and the TCR. Moreover, structure-based approaches could yield predictions that will be applicable to all types of MHC and TCR receptors, mitigating the limitations of small training datasets for rare MHC alleles, which are required for machine learning-based predictions.

Despite the slow progress in the development of structure-based approaches due to the need for serious computational resources and high-resolution models, some attempts in this direction were made. In 2000 Schueler-Furman et al. [166] developed an approach utilizing a pairwise potential matrix that can be applied to a wide range of MHC I molecules for predicting peptide binding. In the following years, new algorithms for the prediction of peptide-MHC complexes binding were developed. PePSSI (peptide-MHC prediction of structure through solvated interfaces) [167] is an approach that allows predicting the structure of peptides bound to HLA-A2. It includes a sampling of peptide backbone conformations and flexible movement of MHC side chains and can explicitly take water molecules at the pMHC interface into account. Initially, PePSSI was tested to predict the conformation of eight peptides bound to HLA-A2, for which crystallography data are available. Analysis of predicted structures in comparison with structures derived from X-ray models showed them to be in good agreement. In [168] a method based on molecular dynamics simulations and estimation of free energy of binding between peptides and HLA molecules was proposed. Another approach, HLAffy, is based on the strength of a mechanistic model of peptide-HLA recognition [169]. It can predict epitopes for any class I HLA by assessing the binging affinity of peptide-HLA complexes by learning pair potentials that are important for peptide binding. Notably, this list of methods and descriptions of structure-based approaches is not exhaustive. For a more comprehensive review of this topic, please refer to [162].

As was mentioned above, some neoantigens that have a high binding affinity to MHC will not be effectively recognized by the TCR [170,171], which makes them unable to trigger T cell-mediated immune response. This fact allows us to suggest the existence of some peptide features that determine their recognition by the TCR independently of MHC binding. In recently published works, it was reported that immunogenic peptides are enriched in hydrophobic and aromatic amino acids at positions interacting with the TCR [172,173]. Other parameters that are believed to influence TCR binding are amino acid charge and bulkiness, WT and mutant sequence divergence and sequence entropy [65,174]. Currently, available tools attempt to solve these challenges by considering these features in the context of the peptide sequence [65,173,174,175]. However, it is evident that the impact of properties such as amino acid charge and size and the composition of hydrophobic residues should be taken into account in the conformation of the peptide bound to the MHC. In this connection, structure-based predictions could be one of the possible ways to determine the impact of physicochemical features of peptides on their immunogenic potential [61,176,177]. In [177], the authors developed a flexible backbone docking protocol called TCRFlexDock utilizing RosettaDock and ZRANK and benchmarked it using 20 structures of TCR/pMHC (17 for MHC class I and 3 for MHC class II) complexes, for which resolved structures of unbound components are available. Testing revealed that protein–protein docking algorithms are able to produce accurate structural models of TCR/pMHC based on unbound component structures [177]. In [176], the authors used a force-field approach utilizing refined versions of FoldX and Rosetta force fields to perform prediction of related targets of the TCR. TCR:p:MHCII complex-based benchmark containing epitope and non-epitope containing pMHC complexes was developed, and immunogenicity was estimated by calculating interaction energies between the TCR and each of the p:MHCII complexes. It was found that the predictive power of this approach depends on the ability to predict protein-MHC complex binding and model the structure of the TCR:p:MHC complex [176]. Riley et al. [61] developed a procedure for accurate and rapid modeling of the structure of nonameric peptides bound with a common class I MHC type HLA-A2 and applied it for analyzing a dataset containing thousands of immunogenic, non-immunogenic and non-HLA2-A2 binding peptides. After that, they trained a neural network (NN) on structural features that affect TCR and peptide binding energies. It was shown that structurally-parameterized NN outperformed other methods that do not include explicit structural or energetic properties in the assessment of CD8^+^ T cell response of HLA-A2 bound nonameric peptides [61]. Thus, a combination of MHC-binding prediction based on NGS-data with a structure-based approach could significantly improve the accuracy of immunogenic peptide selection that is of special importance in the context of peptide-based cancer vaccine development.

## 5. Neoantigen Peptide Databases

A growing number of pan-cancer analyses aimed at neoantigen evaluation make it necessary to consolidate the resulting data into appropriate databases. One of the most potent sources is the IEDB database, that contains data related to immunoepitopes and their identification [65,178,179]. This database is compiled from preliminary data from the literature [178,179]. It has been maintained since 2004 and is currently being actively developed [179]. It also includes an IEDB-AR, a specialized collection of tools for the prediction and evaluation of T and B cell epitopes [180]. In recent years new databases specializing on neoantigen-related data were established (e.g., TANTIGEN, TSNAdb, NeoPeptide, dbPepNeo) [181,182,183,184]. A detailed description of these databases is provided in Table 2.

## 6. Conclusions 

The importance of neoantigens as potential immunotherapeutic agents and prognostic biomarkers is difficult to overstate. The need to further our understanding of neoantigen involvement in the development of tumor-related T cell immune response drives research in this field. It is considered an established fact that tumor mutation burden and, most notably, neoantigen burden exhibit a significant correlation with response to immune checkpoint therapy for certain cancer types (e.g., melanoma, NSCLC, etc.), once again highlighting the effect of neoantigens on the activation of the immune response to tumors. It seems promising to implement these properties of neoantigens in clinical practice. Firstly, estimates of neoantigen prevalence could be utilized as a potential biomarker of response to immune checkpoint inhibitors therapy that has a tremendous relevance, since the number of therapeutic options grows rapidly, and well-defined criteria for developing decision-making algorithms are required. Secondly, it seems logical to apply artificially synthesized tumor-specific neoantigens (e.g., in the form of RNA/DNA or peptide-based vaccines), which are able to activate specific T-cell-mediated immune response leading to tumor degradation. Both applications need an appropriate algorithm allowing accurate and rapid identification of neoantigens specific to individual tumors. To the best of our knowledge, WES and RNA-seq are the starting point for neoantigen discovery approaches that were already used in several trials on model organisms as well as in humans. These methods were implemented in clinical practice owing to the associated significant cost reduction and the development of suitable tools allowing to analyze the generated data. In the context of neoantigen identification, genomic-based approaches potentially allow us to discover almost all types of possible neoantigen sources (with the possible exception of proteasome-generated spliced peptides). However, its application is limited by a lack of evidence about the actual existence of predicted peptides and their ability to be bound and presented by MHC molecules and recognized by the TCR. The development of best practices for the bioinformatic prediction of neoantigens [50] is an important initial point on the way to the unification and standardization of tools and methods that are intended to achieve these goals. Revealing weak points of analysis is useful to equip the current pipeline with new tools that could improve its accuracy by solving the challenges described. It is equally important to note that MS-based methods now are essential because they not only lead to a better understanding of MHC-bound proteome but also help accumulate of data that can be used for improving training datasets and extend the knowledge about the nature of new peptide isoforms (e.g., proteasome-spliced isoforms) and the effect of post-translation modifications on the affinity to the MHC as well as the ability to be recognized by TCRs. Finally, the structure-based prediction could help to overcome limitations of sequence-based approaches, e.g., training datasets for each MHC allele, by adding information regarding the general effects of intrinsic structural and physicochemical properties of peptides on their ability to bind to the MHC and be recognized by the TCR in pMHC complexes.

Authors of this review have a clear understanding that so far the implementation of all the above-mentioned techniques simultaneously for diagnostics purposes (e.g., tumor mutation and neoantigen burden estimation) as well as for the development of personalized neoantigen-based vaccines does not seem realistic for a number of reasons, including the fact that high expenditures and a large highly-qualified team are required to implement all these methods, and a long time is required for collecting and analyzing the generated data and to producing a ready-to-use vaccine. However, at the same time, considering that each approach has its gaps, it is obvious that they cannot predict peptides with absolute certainty and do not guarantee that the predicted peptides are indeed immunogenic. To overcome emerging challenges, it is necessary to integrate efforts of all investigators in this field and create clear and standardized guidelines for the main approaches available (genomics-based, MS-based and structure-based) to make it possible to combine and accumulate generated data, which could potentially improve existing models used in predictions. By including additional high-throughput techniques, such as ribosome profiling approach, and developing tools for solving currently unresolved issues, the current state of the art could be significantly improved.

## Figures and Tables

**Table 2:** Selected databases containing information related to tumor neoantigens *.

Database, Year of Appearance	Source and Description	Refs.
IEDB (The Immune Epitope Database) 2004	Source: https://www.iedb.org/ Description: IEDB is one of the most powerful sources of experimental data concerning immune epitope discovery. It contains information regarding T cell epitopes of human and other organisms. It also provides tools that could be useful for neoantigen prediction. They include MHC class I and II binding predictors, including proteasome cleavage and TAP transport processing steps, as well as tools for immunogenicity predictions. For a review of this topic see [180]	[178,179,180]
TANTIGEN and TANTIGEN 2.0 (Tumor T-cell Antigen Database) 2009	Source: http://projects.met-hilab.org/tadb/ Description: This database contains information about more than 1000 tumor peptides stemming from 292 different proteins. According to the description presented in [181], all peptides in the database are marked as belonging to one of four categories: (1) peptides measured in vitro to bind the HLA, but not reported to elicit either in vivo or in vitro T cell response, (2) peptides found to bind the HLA and to elicit an in vitro T cell response, (3) peptides shown to elicit in vivo tumor rejection, and (4) peptides processed and naturally presented as defined by physical detection. Moreover, peptides are annotated that are naturally processed HLA binders, e.g., peptides eluted from HLA in mass-spectrometry studies. The database also contains predicted binding peptides of 15 HLA class I and Class II.	[181]
TSNAdb 2018	Source: http://biopharm.zju.edu.cn/tsnadb/ Description: TSNAdb is a freely available database developed by Wu et al. [182]. It contains results of somatic mutation identification and HLA typing analysis of 7748 tumor samples of 16 different cancer types obtained from The Cancer Genome Atlas (TCGA) and The Cancer Immunome Atlas (TCIA). Based on this data, the author predicted binding affinity between mutant/wild-type peptides and HLA class I molecules using netMHCpan v2.8/v4.0. Thus, the database contains information about 3707562/1146961 potential antigens.	[182]
NeoPeptide 2019	Source: http://www.neopeptide.cn/ and https://github.com/lyotvincent/NeoPeptide Description: NeoPeptide contains information about neoantigens resulting from somatic mutations gleaned from published literature and immunological resources. As described in [183], it contains 1,818,137 epitopes obtained from more than 36,000 neoantigens that were found in different cancer types (NSCLC, breast cancer, melanoma, etc.) and specifies characteristics such as mutation site, subunit sequence, and MHC complex restriction. The database includes data concerning experimentally characterized epitopes, which are also derived from MHC binding and MHC ligand elution experiments. Information on neoantigens is cited with references to the sources.	[183]
dbPepNeo 2020	Source: http://www.biostatistics.online/dbPepNeo/ Description: dbPepNeo is a manually curated database of experimentally confirmed human tumor antigens that bind specifically to HLA class I, which contains information extracted from peer-reviewed articles and the publicly available data sources. The database relies on mass spectrometry (MS) validation and specific T-cell immunoassays. The peptides were classified according to validation methods: 1. Low confidence (407794): validated by MS only; 2. Medium confidence (247): contain a somatic mutation and are validated by MS and WES/WGS; 3. High confidence (295): immunogenicity was validated directly by utilizing specific T-cell response experiments. dbPepNeo also includes the following tools: ProGeo-neo (see Table 1) and INeo-Epp, a machine learning algorithm for neoepitope immunogenicity prediction using neoantigen peptide features.	[184]

* The information presented here is based on the introductions to these databases provided in the respective articles as well as on details specified on source websites. The database creation date is based on the publication date of the supporting article unless specified otherwise.
